# Differential IL-17A response to *S. pneumoniae* in adenoid tissue of children with sleep disordered breathing and otitis media with effusion

**DOI:** 10.1038/s41598-019-56415-w

**Published:** 2019-12-27

**Authors:** Chien-Chia Huang, Pei-Wen Wu, Ta-Jen Lee, Chyi-Liang Chen, Chun-Hua Wang, Chi-Neu Tsai, Cheng-Hsun Chiu

**Affiliations:** 1 Division of Rhinology, Department of Otolaryngology, Chang Gung Memorial Hospital and Chang Gung University, Taoyuan, Taiwan; 2 Graduate Institute of Clinical Medical Sciences, College of Medicine, Chang Gung University, Taoyuan, Taiwan; 3 Department of Otolaryngology–Head and Neck Surgery, Chang Gung Memorial Hospital and Chang Gung University, Keelung, Taiwan; 4 Molecular Infectious Disease Research Center, Chang Gung Memorial Hospital, Taoyuan, Taiwan; 5 Department of Thoracic Medicine, Chang Gung Memorial Hospital and Chang Gung Memorial Hospital and Chang Gung University, Taoyuan, Taiwan; 6 Division of Pediatric Infectious Diseases, Department of Pediatrics, Chang Gung Memorial Hospital and Chang Gung University, Taoyuan, Taiwan

**Keywords:** Paediatric research, Molecular medicine, Lymphocyte activation

## Abstract

*Streptococcus pneumonia*, one of the major colonizers in nasopharyngeal adenoids, has been the predominant pathogen causing acute otitis media (AOM) in children. Recent evidence suggests an association between IL-17A-mediated immune response and the clearance of pneumococcal colonization in nasopharyngeal adenoids. Here, we evaluated the expressions of IL-17A and associated genes in hypertrophic adenoid tissues of children with sleep-disordered breathing (SDB) and otitis media with effusion (OME) and their association with pneumococcal carriage. Sixty-six pediatric patients with adenoid hypertrophy were enrolled. During adenoidectomy, nasopharyngeal swab and adenoid tissues were used to determine pneumococcal carriage and IL-17A expression. Our results revealed significantly higher levels of IL-17A and IL-17A:IL-10 mRNA in the SDB patients positive for nasopharyngeal pneumococcal carriage than those negative. However, these differences were not significant in the OME group. These results suggested, in OME patients, prolonged or chronic pneumococcal carriage may occur because of insufficient IL-17A-mediated mucosal clearance, and could further lead to AOM and OME development.

## Introduction

Nasopharyngeal adenoids have been speculated to be associated with otitis media, sinusitis, and obstructive sleep apnea syndrome (OSAS) in children^1–4^. The hypertrophic adenoid tissue not only causes narrowing of the upper airway as a common etiology of sleep-disordered breathing (SDB) or OSAS in children^3^, but also represents a reservoir for seeding of pathogens to the middle ear, paranasal sinus, and even the invasive diseases, such as pneumonia, bacteremia, and meningitis^1^. *Haemophilus influenzae, Streptococcus pneumoniae* (*S. pneumoniae*), group A beta-hemolytic *Streptococcus*, *Staphylococcus aureus*, and *Moraxella catarrhalis* are the most common bacteria isolated from nasopharyngeal adenoid^5^. Among them, *S. pneumoniae* is the most common pathogen of acute otitis media (AOM)^6^.

For many mucosal pathogens, pathogenesis begins with the adhesion of microbes to the mucosa, followed likely by transient colonization^1,7^. Bacterial colonization in nasopharyngeal adenoid not only frequently precedes the onset of some invasive diseases but also serves as a reservoir to spread within the community^4,8^. Longitudinal studies in pneumococcus carriage have demonstrated that colonization of the upper airway could be a dynamic process, and most children may gain pneumococcal colonization serially with single or even multiple serotypes^9^. Each episode of colonization might last from days to months, which provides an opportunity for infection. As a result, the ability of host to eradicate the bacteria on the mucosal surface of nasopharynx is very important. Efficient mucosal clearance can reduce or shorten the period of colonization, and may even lower the risk of infection.

Recent studies suggest helper T cells (Th17), regulatory T (T_reg_) cells and their associated cytokines in the nasopharyngeal adenoids play an important role in the response to pneumococcal carriage^10,11^. IL-17A is the key player in the eradication of pneumococcal carriage on the mucosa^12–17^. Balance in the Th17 and T_reg_-mediated immune response determines the pneumococcal carriage in nasopharyngeal adenoid.

Otitis media with effusion (OME) usually occurs in the middle ear of children. The middle ear cavity retains effusion fluid without the symptoms and sign of acute inflammation, like fever or otalgia^18^. The mechanical hypothesis that hypertrophic adenoid may obstruct the orifice of Eustachian tube directly and predispose children to OME is not widely accepted^4^. Recurrent or prolonged infections in the adenoids lead to tubal edema and functional disorders, supporting the reservoir theory^4,8,19^. In clinical practice, adenoidectomy (removal of adenoid) decreases the need for repeated tympanostomy tube placement for OME and recurrent AOM^20^. It has been speculated that decreasing the burden of bacteria residence in the pediatric nasopharynx could reduce or eliminate the probability of retrograde seeding of bacteria via the Eustachian tube to the middle ear^4^. Our previous study investigated the expression level of IL-17A in adenoid tissues of children with SDB and revealed upregulated expression of IL-17A in those with pneumococcal carriage^21^. Here, we further evaluated the expressions of IL-17A and associated genes in the hypertrophic adenoid tissue of children with SDB and OME, and their association with pneumococcal carriage.

## Methods

### Patients

We prospectively recruited children with SDB and OME at Department of Otolaryngology from April 2015 to May 2018. We enrolled patients with following characteristics: (1) age of 3 to 12 years; (2) presenting significant symptoms of OSAS or persistent OME for more than three months; and (3) adenoid hypertrophy scheduled for adenoidectomy (±tonsillectomy or tympanostomy with ventilation tube insertion). We excluded patients with following characteristics: (1) usage of antibiotics during the previous 4 weeks, or (2) congenital anomalies, or (3) major medical disorders or chronic illnesses, such as autoimmune disorders, diabetes, immunodeficiency, nephrotic disease and malignancy.

**Figure 1:**
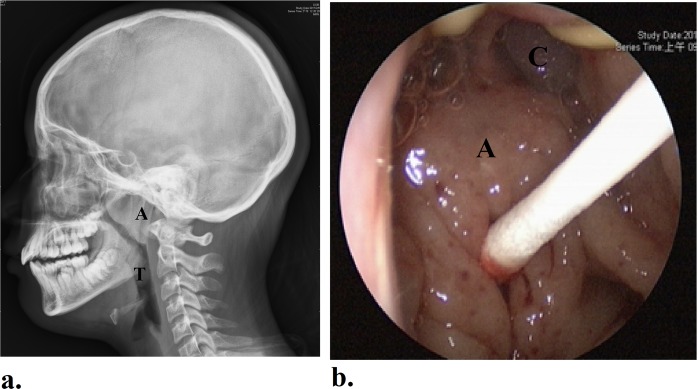
The size of nasopharyngeal adenoids was determined by skull lateral-view radiography (**a**). Nasopharyngeal colonization was determined by swabs for bacterial culture and pneumococcal PCR before removal of adenoid tissue during transoral endoscopic surgery. (**b**) A: adenoid; T: palatine tonsil; C: posterior nasal choanae.

The nasopharyngeal adenoids was measured in size by skull lateral-view radiography preoperatively. The ratio of the length between the outermost point of the anterior convexity of the adenoid tissue and the straight part of the anterior margin of the basicocciput to the length between sphenobasioccipital synchondrosis and the posterior end of the hard palate was defined as Adenoid/Nasopharynx (A/N) ratio which was described by Fujioka *et al*.^22^. (Fig. 1a). OSAS symptom score was evaluated by OSA-18 questionnaire^23^ on the day before surgery and 6 months after surgery.

This study was approved by the Institutional Review Board of Chang Gung Memorial Hospital (103-4773B and 201601090A3C501) and an informed consent was provided by all participants and/or their legal guardians prior to enrollment. All research was performed in accordance with the relevant guidelines and regulations of institutional review board.

### Nasopharyngeal bacterial culture

Bacterial cultures of the surface of the nasopharyngeal adenoid were obtained with sterile swabs before removal of adenoid tissue during transoral endoscopic surgery (Fig. 1b). Swabs in Amies Transport Medium (Copan Italia, Brescia, Italy) were transported to the laboratory and then placed on blood agar plates, Columbia colistin-nalidixic acid agar biplates, and eosin methylene blue plates. The plates was then cultured at 37 °C for 48 h. Bacteriology was determined by colony morphology as well as the conventional methods^24^.

Serotyping of *S. pneumoniae* was determined by multiplex polymerase chain reaction (PCR) using another nasopharyngeal swab, as previously described^25^. In brief, we extract nucleic acids by a QIAamp genomic DNA kit (Qiagen, Valencia, CA, USA) from nasopharyngeal swabs, and kept them frozen at −70 °C until further use. Thirty five primer pairs of specific serotypes were designed, as previously described^25^. Positive control with a primer pair targeting *cpsA* which was found in all 90 known pneumococcal serotypes was used. PCR conditions were as follows: an initial incubation at 94 °C for 4 min, followed by 30 cycles of 94 °C for 45 s, 54 °C for 45 s, and 65 °C for 2 min 30 s. PCR products proceeded to gel electrophoresis on a 1.4% agarose gel at 120 V for 45 min. The gel was then stained with ethidium bromide and visualized by ultraviolet transillumination (Figure S). The oligonucleotide primer sequences was published and available online on the Centers for Disease Control (Atlanta, GA, USA). (http://www.cdc.gov/ncidod/biotech/strep/pcr.htm).

### Adenoidal tissues collection and processing

Adenoidal specimens obtained during transoral endoscopic adenoidectomy were bathed in phosphate-buffered saline (pH 7.6), stored at −70 °C, and then, prepared for real-time PCR.

### Real-time PCR analysis

Total RNA of adenoid tissue was extracted by RNeasy mini kit (Qiagen), quantified, and stained with ethidium bromide to test RNA integrity, according to manufacturer’s instructions as previous decribed^21^. High-capacity cDNA reverse transcription kit (Applied Biosystems, Foster City, CA, USA) and random hexamer primers were used for reverse transcription.

TaqMan assay with target genes specific primer sequences (Table 1) was performed on an Applied Biosystems 7500 fast real-time PCR system (Applied Biosystems) as previous described^21^. The amplification conditions were as follows: 95 °C for 10 min, 45 cycles of 95 °C for 10 s, 60 °C for 20 s, and 72 °C for 10 s, followed with a final cooling period at 40 °C. Triplicate in separate tubes for each sample was performed to allow the quantification of gene expression. Relative mRNA levels of target genes were evaluated by the 2^−∆∆Ct^ method after normalization of the mean threshold cycle (Ct) values to *GAPDH*.

**Table 1:** Primer sequences specific to target genes.

	Forward primers	Reverse primers
IL-12A	TTCACCACTCCCAAAACCTG	AATGGTAAACAGGCCTCCAC
IFN-γ	GCC AAC CTA AGC AAG ATC CCA	ATT TGG AAG CAC CAG GCA TGA
IL-4	TTTGCTGCCTCCAAGAACACA	TCCTGTCGAGCCGTTTCAG
IL-5	AGACCTTGGCACTGCTTTCT	CAGTACCCCCTTGCACAGTT
IL-17A	TTGGTGTCACTGCTACTGCT	TTGGGCATCCTGGATTTCGT
IL-22	AGCCCTATATCACCAACCGC	TCTCCCCAATGAGACGAACG
IL-10	CTCTGATACCTCAACCCCCAT TT	GAGGGAGGTCAGGGAAAACAG
TGF-β	CTG CGG ATC TCT GTG TCA TT	TGC CCA AGG TGC TCA ATA AA
RORγt	CCCAGAACCTCTCTTGGCTT	GAATGGCACAAGTTGGGGTT
FOXP3	AGAGAGCCTGCCTCAGTACA	TGACGCTGCTTCTGTGTAGG
GADPH	TTCCAGGAGCGAGATCCCT	CACCCATGACGAACATGGG

IFN-γ, Interferon gamma; TGF-β, Transforming growth factor beta; RORγt, RAR-related orphan receptor gamma t; FOXP3, Forkhead box P3; GAPDH, glyceraldehyde-3-phosphate dehydrogenase.

### Immunohistochemistry analysis

Paraffin-embedded adenoid tissue sections were de-waxed with xylene, rinsed with absolute alcohol, and bathed in 3% H_2_O_2_ for 30 min for endogenous peroxidase quenching as previous described^21^. The sections were then heated with a microwave for antigen retrieval and incubated in 0.2% normal swine serum (DAKO, CA, USA) for lockage of the positive and negative charges of tissues. Then, the sections went on to one hour incubation with the specific IL-17A antibody (diluted 1:100) or non-specific purified rabbit IgG (diluted 1:100) as a control (GeneTex, CA, USA). Avidin-biotin complex method (LSAB 2 kit; DAKO, CA, and DAB peroxidase substrate kit; Vector Laboratories, Burlingame, CA) was applied for the visualization of antibody labeling.

### Statistical analysis

The statistical analysis was executed with GraphPad Prism 5 software (GraphPad Software, San Diego, CA, USA). Data were presented as mean ± standard deviation. Categorical data was compared using the Chi-squared test. Continuous variables were analyzed by the Mann–Whitney *U* test or unpaired t test when comparing between two groups, as appropriate. Pearson’s correlation coefficient (r) was used to determine the association between two variables. A p-value of <0.05 was considered to be statistically significant. The power was 80.5% as calculating from the difference between the primary outcomes in the study groups.

## Results

### Clinical characteristics of the study population

A total of 66 consecutive pediatric patients with adenoid hypertrophy, including 38 children with SDB who received adenoidectomy + tonsillectomy and 28 children with OME who underwent adenoidectomy + tympanostomy with ventilation tube insertion, were enrolled during the study period. Table 2 summarized the clinical characteristics of the participants. There were no statistical differences between the two groups, except for the pre-operative OSA-18 scores. Pneumococcal carriage was detected in nasopharyngeal adenoids of 14 patients with SDB and 11 patients with OME using either conventional culture or PCR.

**Table 2:** Clinical characteristics of study populations.

	Total	SDB	OME	P value^†^
Case number	66	38	28	
Age (year)	6.4 ± 2.1	6.7 ± 2.1	6.1 ± 2.2	0.188
Male: Female, n	43: 23	27: 11	16: 12	0.241
BMI	17.3 ± 4.1	17.8 ± 4.8	16.5 ± 2.8	1.000
WBC(1000/dL)	8.9 ± 2.8	8.7 ± 2.8	9.1 ± 3.0	0.483
Eosinophil (%)	3.3 ± 2.9	3.4 ± 2.9	3.2 ± 2.8	0.760
Total IgE	347.5 ± 792.6	308.0 ± 569.9	403.2 ± 1048	0.375
A/N ratio (%)	79.0 ± 12.3	79.9 ± 12.1	77.8 ± 12.7	0.405
PCV-7, n (%)	9 (13.6)	6 (15.8)	3 (10.7)	0.553
PCV-13, n (%)	36 (54.5)	18 (47.4)	18 (64.3)	0.173
S. pneumoniae^a^, n (%)	25 (37.9)	14 (36.8)	11 (39.3)	0.840
H. influenzae, n (%)	29 (43.9)	17 (44.7)	12 (42.9)	0.879
M. catarrhalis, n (%)	10 (15.2)	7 (18.4)	3 (10.7)	0.388
S. aureus, n (%)	26 (39.4)	18 (47.4)	8 (28.6)	0.122
OSA-18	66.1 ± 17.8	73.2 ± 16.1	56.6 ± 15.7	<0.001**
Post-op OSA-18	31.2 ± 15.7	29.2 ± 15.0	34.12 ± 16.6	0.211

Data was presented with mean ± SD.

^†^Categorical variables were compared using the Chi-squared test or Fisher’s exact test, as appropriate, and continuous variables were analyzed by the Mann–Whitney U test between SDB and OME groups. **p < 0.01

^a^Determined by either culture or PCR.

SDB, sleep-disordered breathing; OME, otitis media with effusion; BMI, body mass index; A:N ratio, adenoid:nasopharynx ratio; PCV, pneumococcal conjugate vaccine; OSA-18, Obstructive Sleep Apnea-18 questionnaire; post-op, post-operative.

### Real-time PCR analysis

There were no differences in the mRNA levels of IL-12A and interferon gamma (IFN-γ) (Th1-driven cytokines), IL-4 and IL-5 (Th2-driven cytokines), IL-17 and IL-22 (Th17-driven cytokines), and IL-10 and transforming growth factor beta (*TGF-β*) (T_reg_-driven cytokines) between SDB and OME patients (Table 3).

**Table 3:** Expression levels of signature cytokines in patients with adenoid hypertrophy.

	Total (n = 66)	SDB (n = 38)	OME (n = 28)	P value
IL-12A	0.75 ± 0.47	0.77 ± 0.52	0.73 ± 0.40	0.707
IFN-γ	0.87 ± 1.73	1.02 ± 2.25	0.63 ± 0.23	0.739
IL-4	0.85 ± 1.43	0.84 ± 1.20	0.87 ± 1.71	0.473
IL-5	0.75 ± 2.67	0.86 ± 3.47	0.61 ± 0.85	0.581
IL-17A	1.13 ± 0.88	1.23 ± 0.97	0.99 ± 0.73	0.144
IL-22	1.14 ± 4.89	1.65 ± 6.41	0.44 ± 0.55	0.148
IL-10	0.97 ± 1.65	1.11 ± 2.15	0.78 ± 0.25	0.816
TGF-β	0.56 ± 0.52	0.63 ± 0.65	0.46 ± 0.21	0.211
RORγt	0.43 ± 1.36	0.59 ± 1.76	0.20 ± 0.13	0.372
FOXP3	0.66 ± 0.50	0.71 ± 0.59	0.58 ± 0.33	0.485

Data was presented with mean ± SD.

SDB, sleep-disordered breathing; OME, otitis media with effusion; IFN-γ, Interferon gamma; TGF-β, Transforming growth factor beta; RORγt, RAR-related orphan receptor gamma t; FOXP3, Forkhead box P3; GAPDH, glyceraldehyde-3-phosphate dehydrogenase.

**Figure 2:**
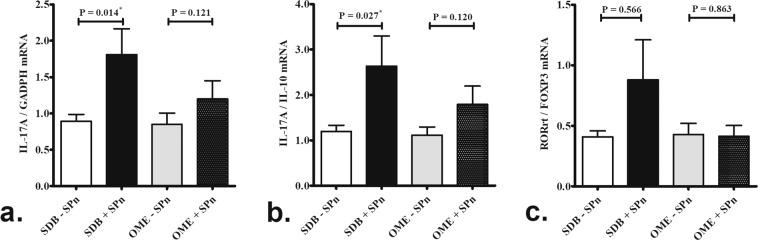
Significantly higher levels of IL-17A mRNA (**a**) and IL-17A:IL-10 mRNA(**b**) in the SDB patients positive for pneumococcal carriage (n = 14) compared SDB patients negative for pneumococcal carriage (n = 24). However, these difference were not statistically significant between the OME patients positive (n = 11) and negative (n = 17) for pneumococcal carriage (**a**,**b**). There was no significant difference in transcription factor RORrt:FOXP3 mRNA expression between the subgroups(**c**). SDB: sleep-disordered breathing; OME: otitis media with effusion; SPn: S. pneumonia. *p < 0.05 according to Mann-Whitney *U* test.

Levels of IL-17A mRNA (Fig. 2a) and IL-17A:IL-10 (Fig. 2b) mRNA were significantly higher in the 14 SDB patients positive for pneumococcal carriage than the 24 SDB patients negative for pneumococcal carriage. However, these differences were not significant between the OME patients positive and negative for pneumococcal carriage (Fig. 2a,b). There was no difference in transcription factor of Th17 cell, RAR-related orphan receptor-γt (RORγt) and transcription factor of T_reg_ cell, forkhead box P3 (FOXP3), between the subgroups (Fig. 2c).

**Figure 3:**
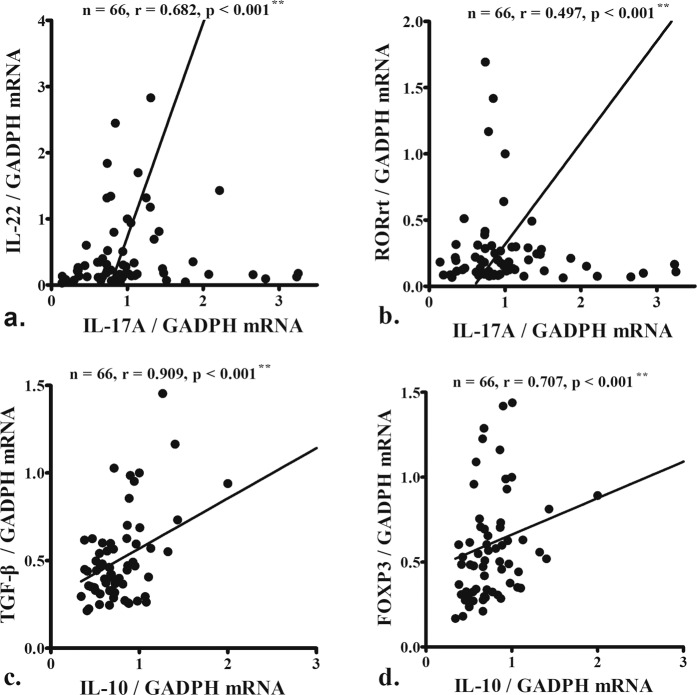
IL-17A mRNA levels correlated with the levels of another Th17 cytokine, IL-22, (**a**) and its transcription factor, RORγt (**b**), whereas IL-10 levels associated with TGF-*β* (**c**) and its transcription factor, FOXP3, levels (**d**). Data were analyzed using Pearson’s correlation coefficient (r). **p < 0.01.

IL-17A mRNA levels correlated with the levels of another Th17 cytokine, IL-22, (Fig. 3a) and its transcription factor, RORγt (Fig. 3b), whereas IL-10 levels associated with TGF-*β* (Fig. 3c) and its transcription factor, FOXP3, levels (Fig. 3d).

### Immunohistochemistry analysis

**Figure 4:**
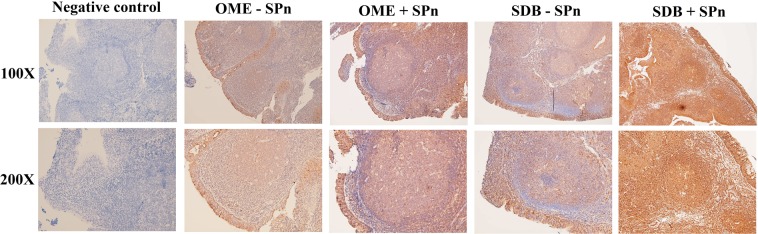
Immunohistochemistry analysis revealed a diffused staining of IL-17A in the epithelium, sub-epithelial tissues, and lymphoid follicles. The difference in IL-17A expression in adenoid tissues between patients positive and negative for pneumococcal carriage was more significant for SDB patients than for OME patients. SDB: sleep-disordered breathing; OME: otitis media with effusion; SPn: S. pneumonia.

Immunohistochemistry analysis revealed a diffused staining of IL-17A in the epithelium, sub-epithelial tissues, and lymphoid follicles. The difference in IL-17A expression in adenoid tissues between patients positive and negative for pneumococcal carriage was more significant for SDB patients than for OME patients (Fig. 4).

## Discussion

The association of Th17-mediated immune response and clearance of pneumococcal colonization in nasopharynx was first proposed by Richard Malley’s study in 2008^12^. They proposed that pneumococcal stimulation on tonsillar cells elicited an IL-17A response. IL-17A could amplify the neutrophils facilitating killing of *S. pneumoniae* both in the absence or presence of antibodies and complement in mice model. Another animal study further demonstrated that efficient clearance of colonization required activation of a population of pneumococcal-specific IL-17-expressing CD4^+^ T cells via toll like receptor 2^13^. Since then, several studies have explored the important role of Th17/T_reg_ cells in responding to pneumococcal colonization^12–17^. For example, Gray *et al*.^14^ suggested a protective task of Th17 against nasopharyngeal pneumococcal colonization. A protein toxin, named pneumolysin, expressed by almost all pneumococcal strains could stimulate Th17 cells of nasopharynx-associated lymphoid tissue in human, which might contribute to eradication of pneumococcal carriage. Jiang *et al*.^15^ reported that downregulation of Th17 cells and upregulation of T_reg_ cells in the adenoids might result in decreased clearance of *S. pneumoniae* and prolonged carriage. Mubarak *et al*.^16^ demonstrated a dynamic relationship between T_reg_ and Th17 populations in nasopharyngeal mucosa. This relationship evolves with age and corresponding to the eradication of pneumococcal carriage. This finding was consistent with another epidemiologic study showing that pneumococcal carriage is prevalent in young children and decreases with age^26^. Hoe *et al*.^17^ reported that increased IL-17 secretion was observed in low density but not high density of pneumococcal-carriage in pediatric nasopharynx. Our previous study revealed an upregulation of IL-17A in SDB patients with pneumococcal carriage^21^.

In the present study, we first evaluated IL-17A expression in adenoid tissue of children with OME and its association with pneumococcal carriage. The results revealed significantly higher levels of IL-17A and IL-17A:IL-10 mRNAs in SDB patients positive for pneumococcal carriage than those negative. However, these differences were not significant between the OME patients positive and negative for pneumococcal carriage. In addition, IL-17A mRNA levels correlated with IL-22 and its transcription factor, RORγt, whereas IL-10 levels were associated with TGF-β and its transcription factor, FOXP3. These results indicate that IL-17A-mediated immune response is associated with pneumococcal carriage in nasopharyngeal adenoids in children with SDB. However, in children with OME, prolonged or chronic pneumococcal carriage may occur because of insignificant response of IL-17A-mediated mucosal clearance and could further lead to AOM and OME development.

Our results in the SDB patients are consistent with Hoe *et al*.'s study showing association between increased IL-17 secretion and low pneumococcal-carriage density. On the other hand, low IL-17 secretion in high pneumococcal-carriage density was similar to that in the OME group in the present study^17^. Our findings also corroborate the study of Gray *et al*., in which they found a higher frequency of Th17 cells in tonsillar mononuclear cells of pneumococcal carriage positive children than carriage negative children before stimulation by pneumolysin. However, the increase in abundance of Th17 cells was more significant in both peripheral blood mononuclear cells and tonsillar mononuclear cells of pneumococcal carriage negative children than carriage positive children after pneumolysin stimulation^14^. IL-17A could be essential for protection from infection in the conditions with low pneumococcal-carriage density. Nevertheless, ineffective or dysregulated IL-17A might provide a favorable environment allowing S. *pneumoniae* to achieve high-density environments. Thereby the opportunities for frequent or chronic infectious diseases increased^17^.

However, our results were inconsistent with those by Jiang *et al*., which demonstrated upregulation of T_reg_ cells and downregulation of Th17 cells in the adenoids of children in pneumococcus-positive groups^15^. Conversely, our data showed increased IL-17A expression in the SDB patients with pneumococcal carriage. Different patient cohorts and different disease settings might have contributed to the differences observed between the studies. In the present study, we comprehensively collected the microbial status, immunologic pattern, as well as clinical information of the enrolled children, which was not mentioned in the other studies. We hypothesize that our results better reflect the actual scenario in clinical practice and have the potential for implementation of new therapeutics targeting IL-17A-mediated immune response.

OME is a common childhood disease and often occurs after AOM; however, it might also occur with Eustachian tube dysfunction in the absence of AOM^18^. Persistent OME can lead to hearing loss that may impair the children’s speech development, school performance, and balance^27^. The pathogenesis of OME, still not fully understood, is considered to be multifactorial, including anatomical, immunological, genetic, microbial, and environmental factors^28,29^. Among them, it is widely accepted that the dysfunction of the Eustachian tube plays a central role in the development of OME at all ages^18^. Nasopharyngeal adenoid serves as a reservoir of microbes, a source of inflammation mediators, and mechanical obstruction leading to tubal dysfunction^27^. Furthermore, *S. pneumonia* is one of the major colonizers in nasopharyngeal adenoid and the most common pathogen of AOM in children^5,6^. Therefore, it is important to define the role of IL-17A-mediated immune pathway in pediatric adenoid and its association with pneumococcal carriage. This study elucidates the possible mechanisms associated with OME and pneumococcal carriage. Future novel therapeutics capable of modifying the balance of Th17/T_reg_ function or direct targeting the IL-17A related inflammatory response might be beneficial for children with persistent OME or recurrent AOM requiring repeated ventilation tube insertion^30,31^.

There were several limitations that warrant consideration in this study. First, this study lacked a normal control group to determine which factor is up- or downregulated. However, it is difficult to recruit appropriate and healthy children because of the ethical considerations involved in performing an invasive biopsy on the adenoid tissue. Instead, we compared our data with previous studies on human and animal models^12–16^. Second, this study implicates an association between pneumococcal carriage and IL-17A. Our finding didn’t provide evidence in associated mechanisms or causative relationships. Finally, large scale studies with more patient groups are needed to investigate the association between IL-17A activity and pneumococcal carriage, and its impact on the clinical outcomes.

## Conclusion

We showed significantly higher levels of adenoidal *IL-17A* and IL-17A:IL-10 mRNA in SDB patients positive for pneumococcal carriage than those negative. However, these differences were not significant in the OME group. These results suggested that prolonged or chronic pneumococcal carriage might occur because of insignificant response of IL-17A-mediated mucosal clearance in OME patients.

## Supplementary information

Figure S.

## Data Availability

All data described in the study has been presented in the manuscript. The datasets analyzed are available from the corresponding author on reasonable request.
