# Supplementary material for: Differential IL-17A response to S. pneumoniae in adenoid tissue of children with sleep disordered breathing and otitis media with effusion
Source: Sci Rep. 2019 Dec 27;9:19839. doi: 10.1038/s41598-019-56415-w (PMC6934741; doi:10.1038/s41598-019-56415-w)
Supplement: Supplementary file 1 — Figure S. [file 41598_2019_56415_MOESM1_ESM.pdf]

Differential IL-17A response to S. pneumoniae in adenoid tissue of children with sleep disordered breathing and otitis media with effusion

Chien-Chia Huang[1,2]; Pei-Wen Wu[1,3]; Ta-Jen Lee[1]; Chyi-Liang, Chen[4]; Chun-Hua Wang[5]; Chi-Neu Tsai[2] & Cheng-Hsun Chiu[4,6]

- [1] Division of Rhinology, Department of Otolaryngology, Chang Gung Memorial Hospital and Chang Gung University, Taoyuan, Taiwan
- [2] Graduate Institute of Clinical Medical Sciences, College of Medicine, Chang Gung University, Taoyuan, Taiwan
- [3] Department of Otolaryngology–Head and Neck Surgery, Chang Gung Memorial Hospital and Chang Gung University, Keelung, Taiwan
- [4] Molecular Infectious Disease Research Center, Chang Gung Memorial Hospital, Taoyuan, Taiwan
- [5] Department of Thoracic Medicine, Chang Gung Memorial Hospital and Chang Gung Memorial Hospital and Chang Gung University, Taoyuan, Taiwan
- [6] Division of Pediatric Infectious Diseases, Department of Pediatrics, Chang Gung Memorial Hospital and Chang Gung University, Taoyuan, Taiwan.

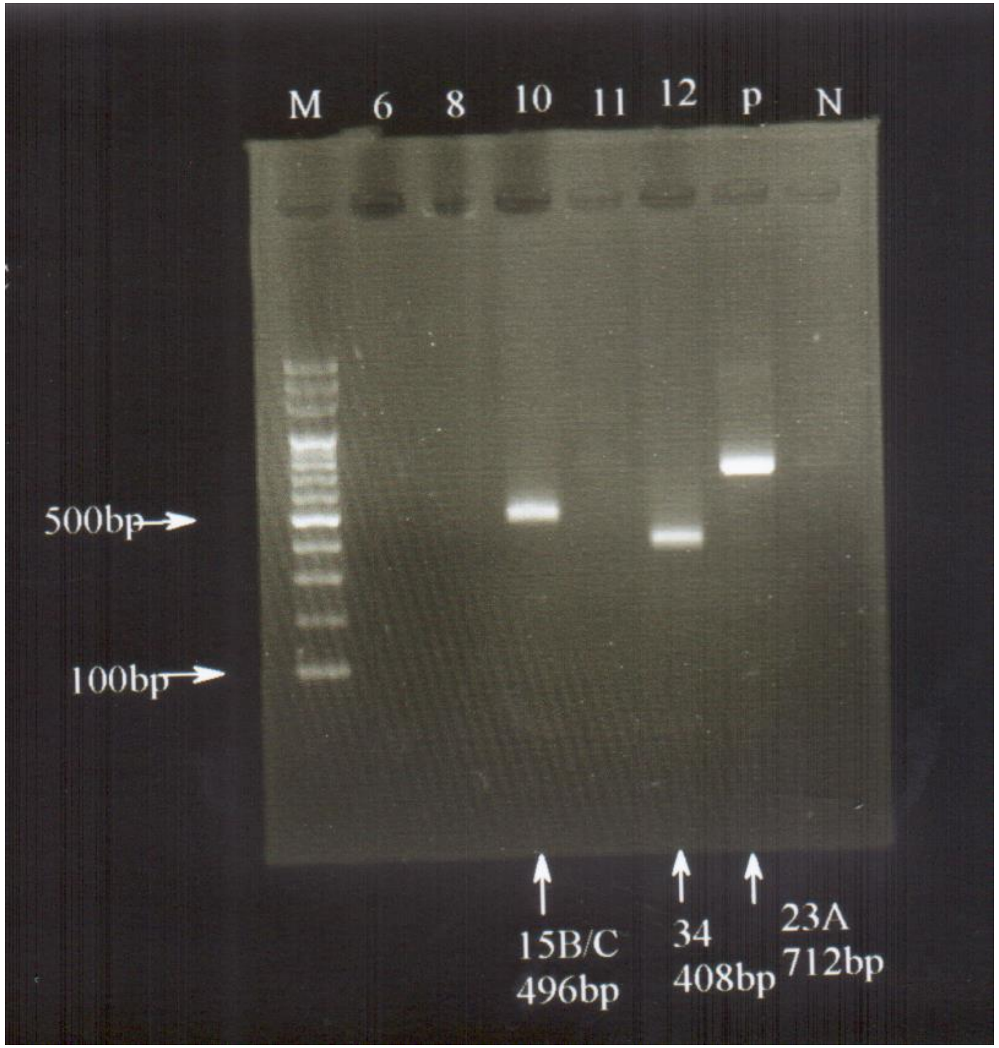

Suppl. Figure S. Multiplex PCR-based serotyping of S. pneumoniae showed the serotypes of sample 10 and 12 were 15B/C and 34 respectively. (single full-length gel)
